# Effects of Adenosine Receptor Antagonists on the In Vivo LPS-Induced Inflammation Model of Parkinson’s Disease

**DOI:** 10.1007/s12640-012-9372-1

**Published:** 2013-01-08

**Authors:** Krystyna Gołembiowska, Jadwiga Wardas, Karolina Noworyta-Sokołowska, Katarzyna Kamińska, Anna Górska

**Affiliations:** Institute of Pharmacology, Polish Academy of Sciences, Smętna 12 Street, 31-343 Kraków, Poland

**Keywords:** Parkinson’s disease, Adenosine A_2A_ receptor antagonists, Inflammation, Oxidative stress

## Abstract

The study shows effects of the nonselective adenosine A_1_/A_2A_ receptor antagonist caffeine and the selective A_2A_ receptor antagonist KW6002 on LPS-induced changes in the extracellular levels of dopamine (DA), glutamate, adenosine, hydroxyl radical, and A_2A_ receptor density in the rat striatum. Intrastriatal LPS (10 μg) injection decreased extracellular level of DA and increased the level of adenosine, glutamate, and hydroxyl radical on the ipsilateral side 24 h after LPS administration. Caffeine (10 and 20 mg/kg i.p.) and KW6002 (1.5 and 3 mg/kg i.p.) given once daily for 6 days and on the 7th day 2 h before and 4 h after LPS injection reversed the LPS-induced changes in extracellular levels of DA, adenosine, glutamate, and hydroxyl radical production. Moreover, LPS-induced decrease in the striatal A_2A_ receptor density was increased by caffeine and KW6002. In order to show the late LPS effect on oxidative damage of DA neurons, the contents of DA, DOPAC, HVA, and hydroxyl radical were determined 72 h after LPS (10 μg) administration into both striata. LPS decreased striatal and substantia nigra content of DA, DOPAC, and HVA while increased striatal but not nigral content of hydroxyl radical. Caffeine (20 mg/kg) and KW60002 (3 mg/kg) given once daily for 6 days and on the 7th day 2 h before and 4 h after intrastriatal injection of LPS normalized the content of DA and its metabolites in both brain regions as well as decreased LPS-induced increase in the striatal level of hydroxyl radical. In conclusion, our data demonstrated antioxidant effects of caffeine and KW6002 in the inflammatory model of PD.

## Introduction

Adenosine A_2A_ receptor antagonists emerged as a new promising non-dopaminergic therapy of Parkinson’s disease (PD) (Schwarzschild et al. 2006). The mechanism of antiparkinsonian effects of A_2A_ receptor antagonists is based on their ability to modulate GABA release and to decrease DA-dependent *c*-*fos* activation in the indirect striatopallidal pathway (Pollack and Fink 1995; Ochi et al. 2000). Presynaptically, A_2A_ receptor antagonists are able to potentiate D_2_ receptor control of glutamatergic transmission which is dysfunctional in PD (Tozzi et al. 2007). A_2A_ adenosine receptor antagonists were shown to alleviate symptoms of PD in a number of behavioral studies in rodents and primates. In a rodent models of PD, A_2A_ adenosine receptor antagonists increased locomotor activity in MPTP-treated or reserpinized mice, reversed haloperidol-induced catalepsy in rats (Shiozaki et al. 1999; Hauber et al. 2001) and potentiated rotational behavior produced by l-DOPA or dopamine agonists in 6-OHDA-lesioned rats (Fenu et al. 1997; Rose et al. 2007). In primates treated with MPTP, the A_2A_ adenosine receptor antagonist istradefylline increased motor activity, decreased dyskinesia induced by a prolonged administration of l-DOPA (Kanda et al. 1998) and produced synergistic effect when added to dopamine agonists (Kanda et al. 2000).

A_2A_ receptors modulate processes accompanying brain injury in animal models of several neurological disorders and recently a neuroprotective potential of A_2A_ receptor antagonists has been suggested (Chen et al. 2007). Epidemiological studies have indicated an inverse relationship between the consumption of caffeine, a non-selective adenosine receptor antagonist, and the risk of developing PD (Ross et al. 2000; Ascherio et al. 2001). The protective effect of caffeine and more selective antagonists of A_2A_ receptors, similar to genetic inactivation of A_2A_ receptors, was observed in an animal MPTP neurotoxicity model (Chen et al. 2007) or in ischemia and excitotoxic brain injury models (Popoli et al. 2004; Chen et al. 2007). The mechanism of neuroprotective action of A_2A_ receptor antagonists is not fully understood but attenuation of overactive glutamate overflow and abatement of oxidative stress may be of importance as shown by several our studies (Gołembiowska et al. 2009; Gołembiowska and Dziubina 2012a, b).

Although etiology of PD is still unclear, it is believed that the progressive degeneration of dopaminergic neurons is associated with chronic neuroinflammation (Dauer and Przedborski 2003; Whitton 2007), and microglia activation is a key factor in this process. Microglial activation is found not only in the vicinity of neurons in the substantia nigra, but also in the putamen, hippocampus, and cortical regions of PD patients (Gerhard et al. 2006; Hirsh and Hunot 2009) as shown in vivo by positron emission tomography. Consistent with the role of inflammation-derived oxidative stress, the brains of PD patients were found to express an increased level of oxidatively modified proteins, upregulation of inducible nitric oxide synthase (iNOS) and cyclooxygenase-2, and decreased activity of glutathione-related genes (Rowe et al. 1998; Knott et al. 2000; Duke et al. 2007). Moreover, in addition to outburst of reactive oxygen species (ROS), the brains of PD patients were observed to contain elevated levels of cytokines and other inflammatory mediators (Whitton 2007). The evidence of ongoing inflammation came also from a number of experimental models. For instance, MPTP treatment in monkeys activated microglia and caused DA neuron loss (McGeer et al. 2003). Similar observations were made in animal models after exposure to toxins, such as MPTP (Członkowska et al. 1996), rotenone (Gao et al. 2003), and 6-OHDA (Mogi et al. 2000).

Microglia cells activated by multiple pro-inflammatory triggers generate ROS, and based on in vitro culture data it is becoming apparent that ROS are the first and essential factor of microglia activation (Gao et al. 2002; Qin et al. 2002). The increase in ROS that occurs in microglia seems to be the response to microglial NADPH oxidase activation that is accompanied by enhanced production of neurotoxic pro-inflammatory factors released from microglia (Qin et al. 2004). Inflammation-related ROS activate astrocytes to release gliotransmitters, in particular glutamate, ATP, and adenosine that may act on adjacent neurons and glia cells to modulate synaptic transmission (Volterra and Meldolesi 2005; Zhang and Haydon 2005). ATP released from astrocytes can suppress neuronal activity after its degradation to adenosine (Dunwiddie and Fredholm 1997). Glutamate release from astrocytes may occur via exocytosis from astrocytic vesicles (Bezzi et al. 2004) or can be mediated by reversal of the glutamate transporters GLAST and GLT-1 (Schousboe and Waagepetersen 2005). Essentially, astrocytes can express receptors for all classical neurotransmitters, modulators, peptides, and cytokines (Verkhratsky and Butt 2007), and neurotransmitters released from presynaptic terminals of neurons can activate these receptors. Similarly, neurotransmitter receptors are also expressed on microglia (Verkhratsky and Butt 2007) allowing neurons to influence pathologic processes via microglia. Adenosine receptors expressed in microglia play a dual role. The activation of A_2A_ receptors in microglia cells may stimulate the release of neurotrophic factors (Heese et al. 1997), while their blockade attenuates the synthesis of prostaglandin E2 (Fiebich et al. 1996) and nitric oxide release (Saura et al. 2005), thus contributing to the neuroprotective effect of adenosine. In contrast, the stimulation of adenosine A_1_ receptors expressed in astroglia controls Ca^2+^ cytosolic signaling and induces apoptosis in microglia culture (Ogata and Schubert 1996), while astrocytic A_2A_ receptor activation inhibits LPS-induced NO production and iNOS expression in C6 glial cells (Brodie et al. 1998). In addition, A_2A_ receptor stimulation regulates expression of glutamate GLT-1 transporter in microglia and astroglia (Li et al. 2001; Nishizaki et al. 2002).

The aim of this study was to investigate in vivo the role of A_2A_ receptors in neuroinflammation induced by intrastriatal LPS injection and related hydroxyl radical production in the rat striatum which is the region with high density of adenosine A_2A_ receptors. The effects of the nonselective A_1_/A_2A_ adenosine receptor antagonist caffeine and the selective A_2A_ receptor antagonist KW6002 on LPS-induced changes in the extracellular levels of DA, glutamate and adenosine, and striatal A_2A_ receptor density were also tested.

## Materials and Methods

### Animals

The study was conducted on male Wistar rats (250–300 g), Charles River, Hanover, Germany. The rats were housed in temperature- and humidity-controlled rooms on a 12-h light/dark cycle, with free access to water and standard pelleted laboratory chow throughout the study. The experimental procedures and housing conditions used were in strict accordance with the Polish legal regulations concerning experiments on animals (Dz. U. 05.33.289). All the experimental protocols were approved by the Local Bioethics Commission for Animal Experiments.

### Drugs

Caffeine and lipopolysaccharide (LPS, Serotype 026:B6) were obtained from Sigma-Aldrich (Poznań, Poland). All the chemicals used for HPLC were purchased from Merck (Warsaw, Poland). Caffeine (Sigma-Aldrich, Poznań, Poland) was dissolved in 0.9 % NaCl, while KW6002 was dissolved initially in dimethyl sulfoxide (DMSO) and was then diluted in at least 20 vol. of 0.9 % NaCl.

### Experimental Protocol

In microdialysis experiments, the non-selective adenosine A_1_/A_2A_ receptor antagonist caffeine in doses of 10 and 20 mg/kg or the selective adenosine A_2A_ receptor antagonist KW6002 [(E)-1,3-diethyl-8-(3,4-dimethoxystyryl)-7-methyl-3,7-dihydro-1*H*-purine-2,6-dione] in doses of 1.5 and 3 mg/kg were given intraperitoneally (i.p.) once daily for 6 days. On the 7th day both drugs were given 2 h before and 4 h after intrastriatal injection of LPS. LPS in a dose of 10 μg dissolved in PBS was infused into left rat striatum in a vol. of 4 μl through combination microdialysis probes (IBR-4, BAS, USA) 24 h before microdialysis. The brains of the animal sub-group subjected to microdialysis were used to test [^3^H]-CGS21680 binding.

In order to show the late LPS effect on oxidative damage of dopamine cells, striatal, and substantia nigra contents of DA, DOPAC, HVA, and hydroxyl radical were determined 72 h after LPS administration. In these experiments, LPS (10 μg) dissolved in 4 μl of PBS was administered into each striatum 72 h before decapitation using a steel needle connected through Teflon tubing with 10 μl Hamilton syringe. Caffeine (20 mg/kg) and KW6002 (3 mg/kg) were injected i.p. once daily for 6 days. On the 7th day both drugs were given 2 h before and 4 h after intrastriatal injection of LPS. Tissue hydroxyl radical content was measured after injection of sodium salicylate (100 mg/kg, i.p.) to rats 20 min before decapitation. All control animals received intraperitoneally respective volumes of vehicles and intrastriatal infusions of PBS.

### In Vivo Microdialysis

The rats were anaesthetized with ketamine (75 mg/kg i.m.) and xylazine (10 mg/kg i.m.) and placed in a stereotaxic apparatus (David Kopf Instruments, Tujunga, CA, USA). Their skulls were exposed and small holes were drilled for the insertion of combination microdialysis probes (IBR-4, BAS, USA) into left striatum using the following coordinates: 1.2 mm anterior from the bregma; 2.8 mm lateral from the sagittal suture; –7.0 mm ventral from the dura (Paxinos and Watson 1998). On the next day, probe inlets were connected to a syringe pump (BAS, IN, USA) which delivered an aCSF composed of [mM]: NaCl 147, KCl 4.0, MgCl_2_ 1.0, CaCl_2_ 2.2; pH 7.4 at a flow rate of 2 μl/min. All metal parts of the aCSF delivery system were replaced with PEEK components or were passivated with 6 M HNO_3_. After 1 h of the washout period dialysate samples were collected every 30 min for 180 min. At the end of the experiment, the rats were sacrificed and their brains were histologically examined to validate probe placement.

### Analytical Procedure

DA was analyzed by high performance liquid chromatography (HPLC) with an electrochemical detection. The level of hydroxyl radicals was estimated as 2,3-dihydroxybenzoic acid (2,3-DHBA), a stable product of the spin trap reagent salicylic acid (0.3 mM) applied via the microdialysis probe. Chromatography was performed using a Dionex P580 pump (USA), an LC-4C amperometric detector with a cross-flow detector cell (BAS, IN, USA) and a Hypersil GOLD C18 analytical column (3 × 100 mm, 3 μm; Thermo Electron Corp., UK). The mobile phase consisted of 0.1 M KH_2_PO_4_ adjusted to pH 3.7 with ortho-phosphoric acid, 0.5 mM EDTA, 20 mg/L 1-octanesulfonic acid sodium salt, and a 3.0 % methanol. The flow rate was 0.7 mL/min, and the applied potential of a 3-mm glassy carbon electrode was +600 mV at a sensitivity of 2 nA/V. Concentrations of all compounds were calculated by comparing their peak areas with respective standards and were processed by Chromax 2001 (Pol-Lab, Warsaw, Poland) software run on a personal computer. The obtained values were not corrected for in vitro probe recovery, which was approximately 10–15 %.

Glutamate was measured in dialysates (20 μL) after its reaction with 4-dimethylaminoazo-benzene-4′-sulfonylchloride (DABS-Cl) at 70 °C for 12 min, according to Knecht and Chang (1986). Dabsylated amino acids were separated on an Ultrasphere ODS (4.6 × 150 mm, 3 μm) column (Supelco, Poznań, Poland) by gradient elution, with solvent A (10 mM citric acid, 4 % dimethylformamide) and solvent B (acetonitrile). Dabsylated compounds were detected by measuring an absorbance at 436 nm using Beckman Amino Acid System Gold with VIS detection.

Adenosine was measured after its reaction with chloroacetal aldehyde to form etheno-adenosine which was assayed by HPLC with fluorescence detection. To 30 μL of dialysate fractions 12 μL of chloroacetal aldehyde and 12 μL of 1 M sodium acetate were added. The mixture was incubated at 80 °C for 20 min and etheno-derivatives of adenosine were separated on C18 Hypersil GOLD column (3 × 100 mm, 3 μm; Thermo Electron Corp., UK). The mobile phase consisted of 0.05 M sodium acetate, pH 6.0, 35 mg/L 1-octanesulfonic acid sodium salt, 5.1 % acetonitrile. Adenosine derivatives were detected at 400 nm extinction and 280 nm emission using RF-2000 fluorescence detector (Dionex, USA). Samples were processed by Chromeleon v. 6.80 software (Dionex, USA) run on personal computer.

### Determination of Tissue Content of DA, DOPAC, HVA, and Hydroxyl Radical in the Rat Striatum

For measurement of DA, 3,4-dihydroxyphenylacetic acid (DOPAC), homovanillic acid (HVA) and hydroxyl radical tissue content, rats were decapitated, their brains were dissected out and the striatum and substantia nigra were separated on ice. Tissue samples were weighted and homogenized in ice-cold 0.1 M perchloric acid. Then, homogenates were centrifuged at 10,000×*g*, supernatants were filtered through membrane filters (0.1 μm pore size) and were injected into the HPLC system. Chromatography was performed using a LC-10 AD pump (Shimadzu Europa GmbH, Warsaw, Poland), an LC-4B amperometric detector with a cross-flow detector cell (BAS, IN, USA), and HR-80 C18 analytical column (4.6 × 100 mm, a 3 μm, ESA, USA). The mobile phase was composed of 0.1 M potassium dihydrogen phosphate (adjusted to pH 3.8 with ortho-phosphoric acid), 0.5 mM EDTA, 80 mg/L 1-octanesulfonic acid sodium salt, and a 4 % methanol. The flow rate was 1 ml/min, and the applied potential of 3 mm glassy carbon electrode was +600 mV with a sensitivity of 5 nA/V. The chromatographic data were processed by Chromax 2005 (Pol-Lab, Warszawa, Poland) software run on a personal computer.

### Autoradiography of [^3^H]-CGS21680 Binding to Adenosine A_2A_ Receptors in the Rat Striatum

The binding of [^3^H]-CGS21680 to adenosine A_2A_ receptors was assayed as previously described (Johansson and Fredholm 1995). In brief, after decapitation, brains were rapidly removed, frozen in cold heptane (−70 °C) and cut into 10 μm coronal sections using a cryostat microtome at −20 °C. The sections were then thaw-mounted on gelatin-coated microscopic slides. Tissue sections were pre-incubated in 170 mM Tris–HCl buffer (pH 7.4) containing 1 mM EDTA and 2 U/mL adenosine deaminase at 37 °C for 30 min. Sections were then washed twice for 5 min at room temperature in 170 mM Tris–HCl buffer with 10 mM MgC1_2_. Incubations were performed for 2 h at room temperature in 170 mM Tris–HCl buffer (pH 7.4) containing the [Carboxyethyl-^3^H(N)]-CGS21680 at the concentration of 6 nM (specific activity 39.0 Ci/mmol, Perkin Elmer Inc, USA), 2 U/mL of adenosine deaminase (Sigma-Aldrich, Poland) and 10 mM MgCl_2_. The non-specific binding was defined on consecutive sections in the presence of 30 μM NECA (5′-*N*-ethylcarboxamido adenosine, Sigma-Aldrich, Poland). The sections were then washed three times for 3 min each in ice-cold Tris–HCl buffer (pH 7.4) at 0 °C, dipped quickly two to three times in ice-cold distilled water and dried at room temperature. The dried tissue sections were then exposed to Tritium Imaging Plate (Fujifilm Europe, GmbH, Poland) for 5 days at room temperature. Calibrated [^3^H] tritium standards (American Radiolabeled Chemicals, Inc., USA) were simultaneously exposed with tissue sections. The plate was scanned using the Fujifilm BAS-5000 phosphorimager. Signal density (the mean optical density per mm^2^ of the ROI) was measured on both sides of the tissue slices in the scanned images using Multi Gauge 3.0 program (Fujifilm Europe, GmbH, Poland). The non-specific binding, which was very low at the level of background, was subtracted for all sections. The binding was estimated at the level *A* = 1.70–1.20 mm from the bregma, according to the stereotaxic atlas of Paxinos and Watson (1998). The data were expressed in fmol/mg of wet tissue as the mean ± SEM for *n* = 6 animals in each group.

### Data Analysis

All obtained data are given in absolute numbers. The statistical significance of differences between experimental groups was calculated using a one-way ANOVA followed by Tukey’s post hoc test. The results were considered statistically significant at *P* < 0.05.

## Results

### The Effect of Repeated Injections of Caffeine and KW6002 on LPS-Induced Changes in the Extracellular Levels of DA, Adenosine, Glutamate, and Hydroxyl Radical Generation in the Rat Striatum

**Fig. 1:**
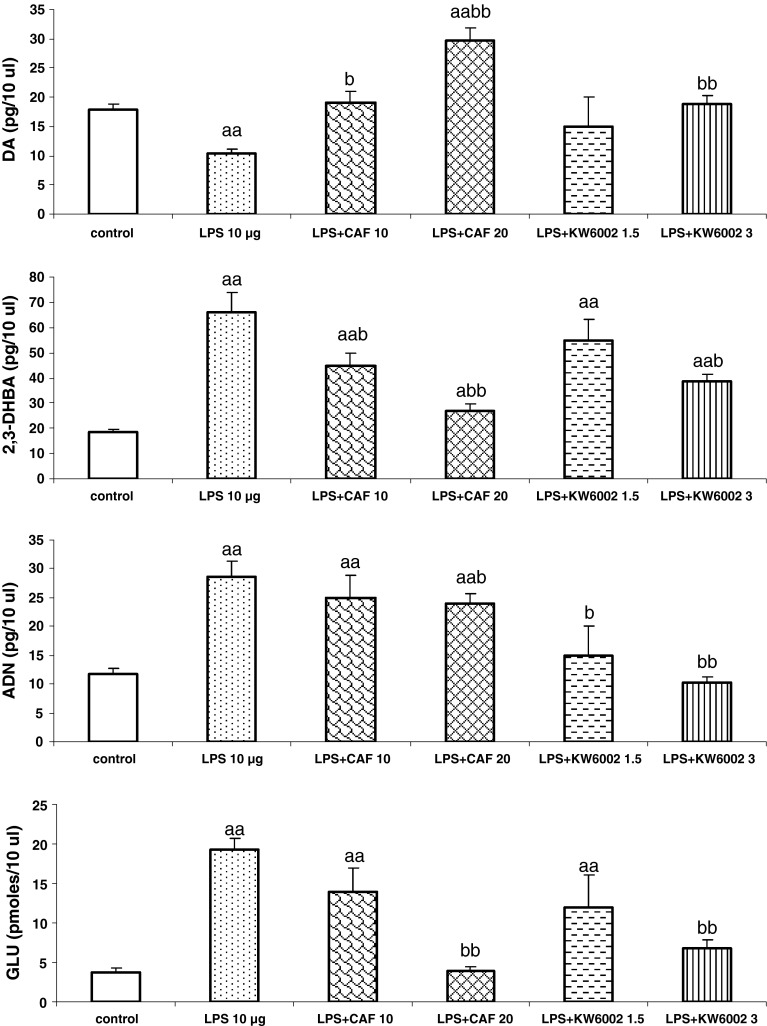
The effect of repeated injections of caffeine and KW6002 on changes in the extracellular levels of dopamine (DA), adenosine (ADN), glutamate (GLU), and hydroxyl radical (estimated as 2,3-DHBA) induced by 10 μg of LPS. LPS was given into the left striatum 24 h before microdialysis experiment. Caffeine (CAF, 10 and 20 mg/kg) and KW6002 (1.5 and 3 mg/kg) were given once daily for 6 days and 2 h before and 4 h after intrastriatal injection of LPS on the 7th day. Values are the mean ± SEM of dialysate fractions collected from 30 to 180 min of experiment, *n* = 6–10 animals per group. ^a^ *P* < 0.05, ^aa^ *P* < 0.001 in comparison to the control group; ^b^ *P* < 0.05, ^bb^ *P* < 0.001 in comparison to LPS (one-way ANOVA and Tukey’s post hoc test)

LPS infusion into the rat striatum at a dose of 10 μg in a volume of 4 μl PBS significantly decreased the extracellular level of DA and increased the extracellular levels of adenosine, glutamate and hydroxyl radical generation in the ipsilateral striatum 24 h after its administration (Fig. 1). The mean DA level in dialysate measured every 30 min from 30 to 180 min of experiment was lowered by ca. 42 % [*F*_1,16_ = 30.33, *P* = 0.00003] by LPS. The extracellular adenosine, glutamate and hydroxyl radical levels were significantly increased by LPS [*F*_1,13_ = 13.96, *P* = 0.001; *F*_1,16_ = 200, *P* = 0; *F*_1,17_ = 7.91, *P* = 0.001, respectively] (Fig. 1).

The LPS-induced decrease in the mean striatal DA level in the ipsilateral striatum was significantly reversed to the control level by KW6002 (3 mg/kg) given repeatedly [*F*_1,19_ = 77.84, *P* = 0] (Fig. 1). The effect of a lower dose of KW6002 (1.5 mg/kg) on LPS-induced decrease in DA extracellular level was not significant. Caffeine (10 mg/kg) administered repeatedly increased DA content to the control level, while at a dose of 20 mg/kg caffeine increased DA level three-fold as compared to the value after LPS treatment and nearly doubled it as compared to the control value [*F*_1,13_ = 4.32, *P* = 0.01; *F*_1,19_ = 276, *P* = 0, respectively] (Fig. 1). The increase in the extracellular adenosine induced by LPS was significantly attenuated by repeated doses of caffeine at a dose of 20 mg/kg, but not by the lower one [*F*_1,16_ = 24.76, *P* = 0.00004]. KW6002 at both doses (1.5 and 3 mg/kg) given repeatedly was effective in lowering LPS-induced increase in extracellular adenosine [*F*_1,13_ = 2.85, *P* = 0.02; *F*_1,16_ = 72.14, *P* = 0, respectively] (Fig. 1). The LPS-induced increase in the extracellular glutamate level was significantly decreased to nearly control value by caffeine (20 mg/kg) [*F*_1,13_ = 203, *P* = 0] and KW6002 (3 mg/kg) [*F*_1,13_ = 25.00, *P* = 0.0002] given repeatedly (Fig. 1). The effect of a lower dose of caffeine (10 mg/kg) and KW6002 (1.5 mg/kg) was not significant in lowering the LPS-induced increase in glutamate level (Fig. 1). The enhancement in hydroxyl radical generation by LPS was suppressed by caffeine at doses 10 and 20 mg/kg [*F*_1,13_ = 2,21, *P* = 0.05; *F*_1,18_ = 5.68, *P* = 0.001, respectively] as well as by KW6002 at a dose of 3 mg/kg [*F*_1,18_ = 5.54, *P* = 0.001], but not at a lower one (Fig. 1). Caffeine (20 mg/kg) and KW6002 (3 mg/kg) given repeatedly to naive rats did not affect the extracellular levels of DA, glutamate, adenosine, and hydroxyl radical production 24 h after treatment cessation (results not shown).

### The Effect of Repeated Injections of Caffeine and KW6002 on LPS-Induced Changes in DA, DOPAC, HVA Contents, and Hydroxyl Radical Production in the Rat Striatum

**Fig. 2:**
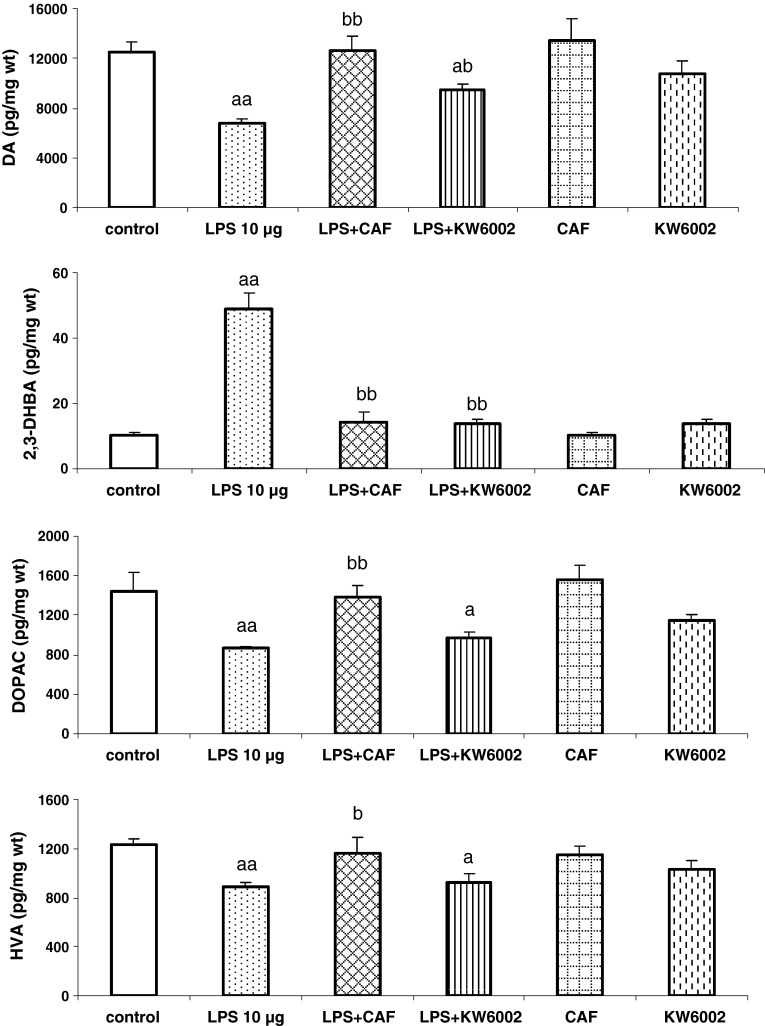
The effect of repeated injections of caffeine and KW6002 on LPS-induced changes in the tissue content of DA, DOPAC, HVA, and hydroxyl radical (estimated as 2,3-DHBA) production in the rat striatum. LPS was given intrastriatally 72 h before decapitation of rats. Caffeine (CAF, 20 mg/kg) and KW6002 (3 mg/kg) were given once daily for 6 days and 2 h before and 4 h after intrastriatal injection of LPS on the 7th day. Values are the mean ± SEM, *n* = 7–10 animals per group. ^a^ *P* < 0.01, ^aa^ *P* < 0.001 in comparison to the control group; ^b^ *P* < 0.05, ^bb^ *P* < 0.001 in comparison to LPS (one-way ANOVA and Tukey’s post hoc test)

LPS (10 μg) given into both sides of the rat striatum 72 h before decapitation of rats induced a decrease in the tissue content of DA [*F*_1,19_ = 34.95, *P* = 0] and its metabolites, DOPAC and HVA [*F*_1,19_ = 84.10, *P* = 0; *F*_1,19_ = 30.65, *P* = 0.00001]. At the same time, a marked increase in the tissue level of hydroxyl radical was observed [*F*_1,19_ = 53.68, *P* = 0] (Fig. 2). Repeated administration of caffeine (20 mg/kg) and KW6002 (3 mg/kg) reversed the decrease in the striatal tissue DA level [*F*_1,19_ = 35.76, *P* = 0.00001; *F*_1,19_ = 27.53, *P* = 0.00003, respectively] and the increase in the striatal tissue hydroxyl radical production induced by LPS [*F*_1,16_ = 28.75, *P* = 0; *F*_1,16_ = 44.13, *P* = 0, respectively] (Fig. 2). Caffeine but not KW6002 increased DOPAC [*F*_1,17_ = 24.46, *P* = 0.0001] and HVA [*F*_1,17_ = 6.55, *P* = 0.02] striatal content, lowered by prior LPS injection (Fig. 2). Caffeine and KW6002 given repeatedly for 7 days to naive rats produced no effect on the striatal content of DA, DOPAC, HVA, and production of hydroxyl radical (Fig. 2).

### The Effect of Repeated Injections of Caffeine and KW6002 on LPS-Induced Changes in DA, DOPAC, HVA Content, and Hydroxyl Radical Production in the Rat Substantia Nigra

**Fig. 3:**
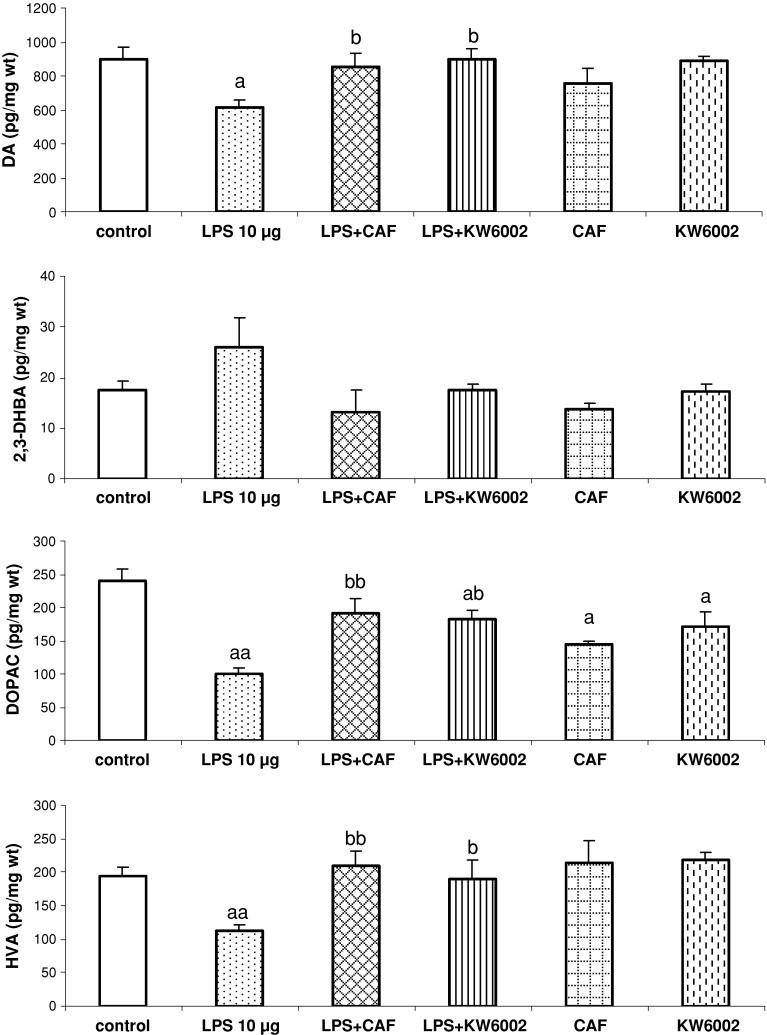
The effect of repeated injections of caffeine and KW6002 on LPS-induced changes in the tissue content of DA, DOPAC, HVA, and hydroxyl radical (estimated as 2,3-DHBA) production in the rat substantia nigra. LPS was given intrastriatally 72 h before decapitation of rats. Caffeine (CAF, 20 mg/kg) and KW6002 (3 mg/kg) were given once daily for 6 days and 2 h before and 4 h after intrastriatal injection of LPS on the 7th day. Values are the mean ± SEM, *n* = 7–10 animals per group. ^a^ *P* < 0.01, ^aa^ *P* < 0.001 in comparison to the control group; ^b^ *P* < 0.05, ^bb^ *P* < 0.001 in comparison to LPS (one-way ANOVA and Tukey’s post hoc test)

Intrastriatal injections of LPS significantly decreased the tissue content of DA, DOPAC and HVA [*F*_1,17_ = 9.50, *P* = 0.007; *F*_1,17_ = 34.42, *P* = 0.00002; *F*_1,17_ = 20.40, *P* = 0.0003, respectively], but had no effect on hydroxyl radical in the rat substantia nigra (Fig. 3). The repeated administration of caffeine (20 mg/kg) and KW6002 (3 mg/kg) reversed the decrease in contents of DA [*F*_1,16_ = 7.73, *P* = 0.02; *F*_1,16_ = 13.52, *P* = 0.02, respectively], DOPAC [*F*_1,16_ = 17.93, *P* = 0.001; *F*_1,16_ = 25.23, *P* = 0.0004, respectively] and HVA [*F*_1,16_ = 20.53, *P* = 0.0006; *F*_1,16_ = 9.43, *P* = 0.01, respectively] induced by LPS, but both drugs had no effect on hydroxyl radical production in the rat substantia nigra. Caffeine and KW6002 did not affect DA and HVA content in the substantia nigra of naive rats 72 h after treatment cessation, but both drugs lowered the tissue content of DOPAC [*F*_1,14_ = 11.15, *P* = 0.005; *F*_1,14_ = 4.66, *P* = 0.05, respectively] (Fig. 3).

### The Effect of Repeated Injections of Caffeine and KW6002 on LPS-Induced Changes in [^3^H]-CGS21680 Binding to Adenosine A_2A_ Receptors in the Rat Striatum

**Fig. 4:**
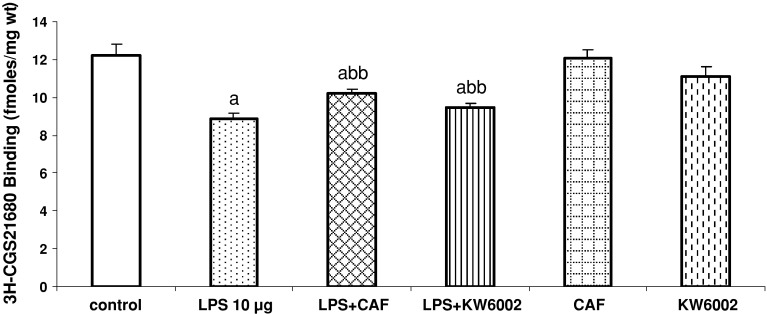
The effect of repeated injections of caffeine (CAF, 20 mg/kg) and KW6002 (3 mg/kg) on LPS-induced changes in [^3^H]-CGS21680 binding to adenosine A_2A_ receptors in the rat striatum. Values are the mean ± SEM, *n* = 6 animals per group. ^a^ *P* < 0.01 in comparison to the control group; ^bb^ *P* < 0.001 in comparison to LPS (one-way ANOVA and Tukey’s post hoc test)

The specific binding of [^3^H]-CGS21680 was reduced (*P* < 0.01) in the striatum after LPS administration [*F*_1,11_ = 22.77; *P* = 0.01] (Fig. 4). Caffeine (20 mg/kg) and KW6002 (3 mg/kg) given repeatedly inhibited LPS-induced decrease in the [^3^H]-CGS21680 binding [*F*_1,11_ = 25.07, *P* = 0.001; *F*_1,11_ = 13.23, *P* = 0,001, respectively] (Fig. 4). The non-specific binding for [^3^H]-CGS21680 was at the background level and was subtracted from the total binding.

## Discussion

The results of our study showed that intrastriatal injection of LPS enhanced the extracellular production of hydroxyl radical accompanied by an increased extracellular level of glutamate and adenosine in the rat striatum 24 h after the treatment. An increased production of hydroxyl radical was still evidenced in the rat striatum but not in the substantia nigra 72 h after LPS administration. The damaging effect of LPS-induced hydroxyl radical production on DA cells was reflected by a decrease in the extracellular DA level and its tissue content not only in the striatum where LPS was applied but also in the substantia nigra. The changes induced by LPS in the levels of DA, hydroxyl radical, adenosine, and glutamate were reversed by caffeine and KW6002. Attenuation of deficit in tissue content of DA, DOPAC, and HVA in striatum and substantia nigra by both drugs confirmed neuroprotective effects of caffeine and KW6002. Moreover, caffeine and KW6002 inhibited the LPS-induced decrease in the striatal A_2A_ receptor density.

LPS, a component of the gram-negative bacteria cell wall is a potent inducer of inflammation. When injected intracerebrally, it activates glia cells in vivo and causes secretion of neurotoxins, such as ROS and other pro-inflammatory mediators (Qin et al. 2004). ROS production originating from microglial NADPH oxidase activity or mitochondrial electron transport chain complexes is toxic for DA neurons as shown elsewhere (Qin et al. 2004; Hunter et al. 2007). In our study, LPS-injected intrastriatally increased hydroxyl radical generation, which was observed in the extracellular space 24 h after LPS administration. The enhanced level of hydroxyl radical in the striatal tissue evidenced 72 h after LPS injection indicates the progression of inflammatory reaction and development of oxidative stress. The lack of increase in hydroxyl radical level in the substantia nigra suggests that inflammation-induced oxidative stress was limited to the striatum, i.e., to the region of LPS injection. Nevertheless, neurotoxic effect of LPS was seen either in the striatum or in the substantia nigra as shown by the decreased extracellular level of DA in the striatum as well as decreased content of DA, DOPAC, and HVA in both studied regions. Thus, LPS-induced inflammatory reaction and oxidative stress which occurred in the rat striatum caused also damage of dopamine cell bodies in the substantia nigra. Our observation is supported by the findings of Przedborski et al. (1995), who showed retrograde degeneration of dopamine cells in the substantia nigra after intrastriatal 6-OHDA injection.

ROS generated by LPS activate astrocytes to release gliotransmitters: glutamate, ATP and adenosine (Volterra and Meldolesi 2005). The activated microglia may be another source of glutamate and the breakdown product of ATP, adenosine (Barger et al. 2007; Nakamura 2002). It is known that the physiological extracellular concentration of adenosine is very low. However, in the presence of injurious insults, such as chronic inflammatory conditions, the concentration of adenosine increases dramatically. In our study, adenosine level increased ca. threefold over the control level after LPS. Under physiological conditions, adenosine may exert inhibitory effect on the release of excitatory neurotransmitters, mainly glutamate from neuronal presynaptic terminals or may reduce astrocyte proliferation and the release of nerve growth factors (Haskó et al. 2005). On the other hand, during inflammation, adenosine activates A_2A_ receptor and inhibits glutamate uptake through the glial glutamate transporter GLT-1 in astrocytes or microglia (Nishizaki et al. 2002; Streit and Xue 2009). In turn, glutamate released from astrocytes stimulates neurons and enhances excitatory synaptic transmission via *N*-methyl-d-aspartate receptor-mediated mechanism (Bezzi et al. 2004). In addition, the stimulation of presynaptic A_2A_ receptors by adenosine may increase glutamate release from the cortico-striatal neuronal terminals (Tozzi et al. 2007). Excessively high extracellular glutamate may result in neurodegeneration caused by its excitotoxic action. In our study, intrastriatal injection of LPS caused increase in the extracellular level of glutamate and different sources (glial or neuronal) might contribute to this increase.

In this study, we showed that repeated treatment of rats with the nonselective antagonist of adenosine A_1_/A_2A_ receptor caffeine as well as the selective A_2A_ receptor antagonist KW6002 prevented the LPS-induced increase in the striatal adenosine and extracellular glutamate levels and hydroxyl radical production. These changes seem to be responsible for neuroprotective action of caffeine and KW6002, since both drugs reversed the LPS-induced decrease in the extracellular DA level and its tissue content in the rat striatum 24 and 72 h, respectively, after LPS administration. Caffeine and KW6002 were given to rats repeatedly for 7 days in small doses. In our earlier studies, the selective A_2A_ receptor antagonists CSC and ZM 241385, given acutely or chronically, were effective as neuroprotectants and attenuated free radical generation in animal PD models after administration of 6-OHDA or reserpine (Gołembiowska et al. 2009; Gołembiowska and Dziubina 2012a, b). Caffeine treatment for 14 days restored also the content of monoamines and prevented the reduction in dopaminergic cell viability after intrastriatal injection of 6-OHDA (Aguiar et al. 2006). However, in the present inflammatory model with the use of LPS, single doses of caffeine or KW6002 were not consistent in reversing the changes induced by LPS (results not shown). Moreover, in the current work we wanted to mimic studies which suggested antioxidant potential of caffeine after its chronic use (Noschang et al. 2009). The protective mechanism afforded by caffeine and KW6002 may be related to their ability to inhibit the activation of glial cells and to suppress oxidative stress resulting from glial cell stimulation. Earlier studies demonstrated that caffeine or the more specific A_2A_ receptor antagonist CSC decreased the number of microglia and astrocytes in population of rat mesencephalic cells treated with 6-OHDA or in the hippocampus of rats after exposure to LPS (Brothers et al. 2010; Nobre et al. 2010). In mice, intracerebroventricular administration of KW6002 attenuated MPTP-induced striatal microglial and astroglial activation and exerted neuroprotective effect (Yu et al. 2008). It may be suggested that attenuation of glial cell activity by adenosine A_2A_ receptor antagonists may diminish secretion of various mediators and transmitters from these cells, such as glutamate or ATP, an extracellular substrate of adenosine, as well as may suppress ROS production. Another likely mechanism of diminution of glutamate release involves blockade of glial GLT-1 transporter by adenosine A_2A_ antagonists and clearance of synaptic glutamate by astrocytes (Pintor et al. 2004). The damaging effect of glutamate resulting in excitotoxicity and oxidative stress can be also caused by striatal presynaptic A_2A_ receptor activation by adenosine. Therefore, reduction of the extracellular adenosine level by A_2A_ receptor blockade shown in our present study may also account for the protective effects of A_2A_ antagonists.

Some recent reports have demonstrated that neuroprotection elicited by A_2A_ antagonists may result from their antioxidant effects. Caffeine and CSC prevented lipid peroxidation in the cytotoxicity model induced by 6-OHDA (Nobre et al. 2010). Chronic administration of caffeine increased activity of antioxidant enzymes: superoxide dismutase, glutathione peroxidase and catalase in several regions of the rat brain (Noschang et al. 2009). Furthermore, caffeine, which is structurally similar to adenosine and to a final product of purine metabolism in humans, uric acid, increased glutathione synthesis in the hippocampus of mice leading to neuroprotection (Aoyama et al. 2011). Scavenging properties of caffeine and 1,3,7-trimethyl uric acid, a primary metabolite of caffeine in rodents (Kot and Daniel 2008) were also demonstrated in our earlier work (Gołembiowska et al. 2008). In addition, we showed that attenuation of hydroxyl radical generation in the striatum of rats with altered VMAT function or in rats with nigrostriatal DA neurons damaged by 6-OHDA was responsible for protective effect of the selective A_2A_ receptor antagonists CSC and ZM 241385 (Gołembiowska and Dziubina 2012b). In our current study, we show that in experimental model of neuroinflammation evoked by LPS, caffeine and KW6002 prevent neurodegeneration of DA neurons by attenuation of oxidative stress.

There is evidence that A_1_ receptor desensitization occurs in several pathophysiological conditions and chronic stimulation of these receptors rather exacerbates neuronal loss which limits their usefulness as a target for neuroprotection (Cunha 2005). On the other hand, A_2A_ receptors might be up-regulated by chronic stressful stimuli (Cunha 2005) and A_2A_ receptor blockade confers robust neuroprotection, the more so that A_2A_ receptors might control the process of neuroinflammation. Therefore, in our present study we tested possible changes in A_2A_ receptor density in the striatum of rats treated repeatedly with caffeine and KW60002 in the model of LPS-induced neuroinflammation. We found that repeated treatment of rats with caffeine and KW6002 did not affect A_2A_ receptor density in the striatum as demonstrated with [^3^H]-CGS21680 binding in normal animals. However, in the striatum of LPS-treated rats we observed a decrease in [^3^H]-CGS21680 binding in the ipsilateral striatum. It has to be mentioned that in our study [^3^H]-CGS21680 binding reflected density of global population of striatal A_2A_ receptors, so both receptors with neuronal and glial cellular location were counted. Therefore, it may be suggested that the decrease in global density of adenosine A_2A_ receptors by LPS results from damaging effect of hydroxyl radical on cellular proteins, such as membrane receptors. The inhibitory effect of caffeine and KW6002 on LPS-produced decrease in A_2A_ receptor density explains reversal by these drugs of the changes induced by LPS in the level of DA, glutamate, adenosine, and hydroxyl radical. However, our data do not exclude the possibility that neuroprotection observed after treatment with caffeine and KW6002 in the inflammatory model of neurotoxicity may not be mediated by A_2A_ receptor blockade. As mentioned above, caffeine increased glutathione level in the hippocampus of mice by increasing sodium-dependent cysteine uptake (Aoyama et al. 2011) and caffeine increased the activity of antioxidant enzymes, such as superoxide dismutase or catalase (Noschang et al. 2009). So far, there is no data showing antioxidant properties of KW6002 such as enhancement of glutathione synthesis or activity of enzymatic antioxidant system in the brain. Nevertheless, in our study caffeine as well as KW6002 inhibited hydroxyl radical production in the model of LPS-induced neuroinflammation. Another possible mechanism of the antioxidant effect of caffeine and KW6002 is underlain by a potent inhibitory effect of caffeine and its analogues, especially the compounds with the styryl side chain, on monoamine oxidase B (MAO-B) activity (van den Berg et al. 2007). The activity of MAO-B, an enzyme present in the outer mitochondrial membrane of neuronal and glial cells, is increased under demanding metabolic conditions, for instance during inflammation. Given that mitochondria are a significant source of ATP and ROS, caffeine analogs, by inhibiting MAO-B activity, may regulate mitochondrial function, restore energy production needed for synaptic release of neurotransmitters and decrease oxidative stress. The observation that caffeine and KW6002 decreased the content of DOPAC, a product of MAO-B activity in DA metabolism in the striatum and substantia nigra speaks for the hypothesis that DA metabolism via MAO-B may contribute to oxidative stress during inflammation.

In conclusion, the findings of this study clearly show that the nonselective A_1_/A_2A_ adenosine receptor antagonist caffeine and the selective A_2A_ receptor antagonist KW6002 have anti-inflammatory and neuroprotective potential in a rat model of neuroinflammation.
